# Analysis of genetic mutations associated with anti-malarial drug resistance in *Plasmodium falciparum *from the Democratic Republic of East Timor

**DOI:** 10.1186/1475-2875-8-59

**Published:** 2009-04-09

**Authors:** Afonso de Almeida, Ana Paula Arez, Pedro VL Cravo, Virgílio E do Rosário

**Affiliations:** 1 Universidade Nacional de Timor Leste, Avenida Cidade de Lisboa, Díli, Timor Leste; 2 Centro de Malária e Outras Doenças Tropicais, Instituto de Higiene e Medicina Tropical, Universidade Nova de Lisboa, Rua da Junqueira 96, 1349-008 Lisboa, Portugal; 3 Unidade de Biologia Molecular, Instituto de Higiene e Medicina Tropical, Universidade Nova de Lisboa, Rua da Junqueira 96, 1349-008 Lisboa, Portugal

## Abstract

**Background:**

In response to chloroquine (CQ) resistance, the policy for the first-line treatment of uncomplicated malaria in the Democratic Republic of East Timor (DRET) was changed in early 2000. The combination of sulphadoxine-pyrimethamine (SP) was then introduced for the treatment of uncomplicated falciparum malaria.

**Methods:**

Blood samples were collected in two different periods (2003–2004 and 2004–2005) from individuals attending hospitals or clinics in six districts of the DRET and checked for *Plasmodium falciparum *infection. 112 PCR-positive samples were inspected for genetic polymorphisms in the *pfcrt*, *pfmdr1*, *pfdhfr *and *pfdhps *genes. Different alleles were interrogated for potential associations that could be indicative of non-random linkage.

**Results:**

Overall prevalence of mutations associated with resistance to CQ and SP was extremely high. The mutant form of *Pfcrt *(76T) was found to be fixed even after five years of alleged CQ removal. There was a significant increase in the prevalence of the *pfdhps *437G mutation (X^2 ^= 31.1; p = 0.001) from the first to second survey periods. A non-random association was observed between *pfdhfr*51/*pfdhps*437 (p = 0.001) and *pfdhfr *59/*pfdhps *437 (p = 0.013) alleles.

**Conclusion:**

Persistence of CQ-resistant mutants even after supposed drug withdrawal suggests one or all of the following: local *P. falciparum *may still be inadvertently exposed to the drug, that mutant parasites are being "imported" into the country, and/or reduced genetic diversity and low parasite transmission help maintain mutant haplotypes. The association between *pfdhfr*51/*pfdhps*437 and *pfdhfr *59/*pfdhps *437 alleles indicates that these are undergoing concomitant positive selection in the DRET.

## Background

In the Democratic Republic of East Timor (DRET), chloroquine (CQ) resistance in *Plasmodium falciparum *was first reported in the 1980s [1,2]. Despite this, until 1999, CQ plus primaquine continued to be used as the first-line treatment of uncomplicated malaria, with sulphadoxine-pyrimethamine (SP) as second-line, severe malaria being treated with quinine [3].

In 1992, high levels of resistance to chloroquine were reported [4]. *In vitro *and *in vivo *resistance to choroquine, amodiaquine and SP was documented in the district of Lospalos, where more than 67% treatment failure to CQ was reported between 1999 and 2000 [3,5-8]. Consequently, in early 2000, the policy for the first-line treatment of uncomplicated malaria was changed. SP was introduced for the treatment of uncomplicated falciparum malaria and chloroquine was used for the treatment of *Plasmodium vivax *only. Currently, SP has been replaced by artemether-lumefantrine for treating *P. falciparum*, but chloroquine is still the recommended treatment for vivax malaria.

Genetic variation associated with both CQ and SP resistance can be monitored with specific molecular markers. The K76T mutation at the *pfcrt *is considered a reliable genetic marker for CQ resistance. Polymorphisms in *pfmdr*1, which encodes the *P. falciparum *P glycoprotein homologue 1, modulate chloroquine resistance in mutant *pfcrt*-harboring parasites *in vitro *[9], although their role *in vivo *has not been sufficiently substantiated [10].

Molecular mechanisms of antifolate resistance in *P. falciparum *have been explored in detail [11]. Specific point mutations in the parasite's dihydrofolate reductase (*dhfr *and dihydropterate synthase (*dhps*) genes are associated resistance to pyrimethamine-sulphadoxine [12-14]. The first study of SP efficacy for the treatment of uncomplicated falciparum malaria, conducted in 2001, reported 81.6% single *pfdhfr *108N or double C59R/S108N mutants, but none of the isolates harboured mutations in *dhps*, and the drug was confirmed to be efficacious [15].

The aim of the present study was to investigate the proportions and distribution of molecular polymorphisms in the parasite *Plasmodium falciparum *dihydrofolate reductase (*pfdhfr*), dihydropterate synthase (*pfdhps*), chloroquine resistance transporter (*pfcrt*) and multi-drug resistance (*pfmdr*1) genes, from samples collected four to five years after the replacement of CQ by SP as the recommended first-line treatment.

## Methods

### Study area

**Figure 1:**
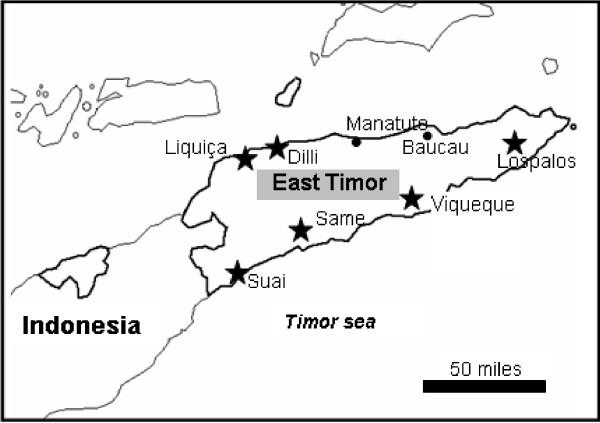
**Map of the Democratic Republic of East Timor showing locations of the districts surveyed**.

The Democratic Republic of East Timor (DRET) is situated on the Eastern Part of the Island of Timor, the eastern most of the Lesser Sunda Island (Figure 1). The study was carried out in two different periods. The first period (2003/2004) was carried out in two districts: (Dili and Suai), and the second period (2004/2005) was carried out in the former and an additional four districts: (Liquiça, Same, Viqueque, Lospalos). These districts can be classified into three different zones, according to geographical location: North (Dili and Liquica), South (Suai, Same and Viqueque) and East (Lospalos).

### Sample collection

Two-hundred and fifty blood samples were collected from 17 November 2003 to 7 January 2004 (period 1) and 650 from 15 December 2004 to 9 March 2005 (period 2). This study was approved by Ministry of Health (Ref.: MS-DG/PESQUISA-IHMT/XI/03/346 and MS/VM/PESQUISA/XII/04) and patients gave their consent to participate. Finger-prick blood samples were collected by passive case detection (PCD) from suspected malaria carriers presenting at hospital/clinic, after informed consent was obtained. No age restrictions were applied. Collected blood was used to make thin and thick Giemsa-stained smears, which were checked for malaria parasites by optical microscopy. A sub-sample of the collected blood was blotted onto filter paper for assessment of molecular markers.

### Genotyping

Genomic DNA was extracted with phenol-chloroform [16] and confirmed to include *P. falciparum *DNA, by use of a species-specific PCR [17]. Each sample was then checked for polymorphisms in three codons (51, 59 and 108) of the *pfdhfr *gene and two codons (437 and 540) of the *pfdhps *gene, using a slight modification of the PCR-RFLP methodology described by [18]. Polymorphisms in two codons (75 and 76) of the *pfcrt *gene and two codons (86 and 1246) of the *pfmdr*1 gene were detected as described previously [19]. Digested products were separated on 2–3% agarose gel, then stained with ethidium bromide and visualized under UV.

### Statistical analysis

A Chi-square (χ^2^) Test was performed to compare differences in frequency of mutant alleles among codons of the *pfcrt*, *pfmdr*1, *pfdhfr *and *pfdhps *genes and between periods of sampling, using SPSS 11.5 for windows, and P < 0.05 was considered significant. Association between alleles at any two loci was tested by Fisher's Exact Test. Data from isolates containing both alleles (polyclonal infections) were excluded from the analysis.

## Results

A total of 900 samples were checked for presence of malaria. Mean age of sampled individuals was 21 ± 17.04 (SD) of which 51% and 49% were males and females, respectively. Prevalence of falciparum, vivax and mixed malaria infections as determined by optical microscopy (OM) was underestimated in all cases, with PCR allowing detection of a significantly higher number of cases (X^2 ^= 15,02; p = 0,001). Thus, infection prevalence detected by OM *vs *PCR, for *P. falciparum*, *P. vivax *and mixed infections, was 7.5% *vs *11.4%, 3.7% *vs *6.6% and 0% *vs *1.0%, respectively.

38 of the 250 and 74 of the 650 patients were infected by *P. falciparum *in period 1 and period 2, respectively, as determined by PCR. Results of Single Nucleotide Polymorphism (SNP) typing are summarized in Table 1. Over 97% of the samples inspected showed the CQ resistance *pfcrt *76 core mutation and of these, 38 and 56% had the additional *pfmdr*1 86Y mutation in the first and second periods respectively. The 75E polymorphism was also observed in 12.5% of isolates from period 2. No mutant alleles at codon *pfmdr*1 1246 were detected.

**Table 1:** Frequency (%) of the *Pfdhfr *(codons 51, 59 and 108), *Pfdhps *(codons 437 and 540), *Pfcrt *(codons 76 and 75), *Pdmdr*1 (codons 86 and 1246) genotypes and multiple mutations in first and second assay periods.

		**Period 1** **(2003–2004)**		**Period 2** **(2004–2005)**	
		
**Gene**	**Codon**	**(N = 38)**	**(%)**	**(N = 74)**	**(%)**
***Pfcrt***	**75**				
	(wild-type) N	16	100	27	56
	(Mutant) E	0	0	6	13
	N + E	0	0	15	31
	Not determined	22		22	
	**76**				
	(wild-type) K	1	3	0	0
	(Mutant) T	32	97	59	100
	K + T	0	0	0	0
	Not determined	5		15	
***Pfmdr1***	**86**				
	(wild-type) N	8	24	18	27
	(Mutant) Y	13	38	37	56
	N + Y	13	38	11	17
	Not determined	4		8	
	**1246**				
	(wild-type) D	32	100	64	97
	(Mutant) Y	0	0	0	0
	D + Y	0	0	2	3
	Not determined	6		8	
***Pfdhfr***	**51**				
	(wild-type) N	9	24	8	11
	(Mutant) I	25	66	66	89
	N + I	4	10	0	0
	Not determined	0		0	
	**59**				
	(wild-type) C	11	29	9	14
	(Mutant) R	24	63	53	84
	C + R	3	8	1	2
	Not determined	0		11	
	**108**				
	(wild-type) S	0	0	2	3
	(Mutant) N	28	100	62	97
	S + N	0	0	0	0
	Not determined	10		10	
***Pfdhps***	**437**				
	(wild-type) A	14	40	0	0
	(Mutant) G	21	60	67	100
	A + G	0	0	0	0
	Not determined	3		7	
	**540**				
	(wild-type) K	34	97	67	100
	(Mutant) E	1	3	0	0
	K + E	0	0	0	0
	Not determined	3		7	
**Multiple mutations**					
	dhfr 51I+108N	19	68	60	97
	dhfr 59R+108N	17	61	52	84
	dhfr 51I +59R+108N	16	57	53	86
	dhfr 51I +59R+108N+dhps 437G	13	46	51	82

More than 97% of the parasite population inspected exhibited the *pfdhfr *core mutation 108N, and an increase in mutant alleles from the first to second period in two others loci examined (*pfdhfr*51 and *pfdhfr *59) was detected. All isolates presented the *Pfdhfr *108N mutation in combination with *dhfr *51I and/or *dhfr *59R. In particular, the *pfdhfr *triple mutation was seen in the great majority of samples (Table 1). There was a significant difference (X^2 ^= 31.1; p = 0.001) in the prevalence of the *pfdhps *437G mutation which increased from 60% to 100% from the first to second periods, respectively. The quadruple mutation [triple *dhfr *(51I, 59R and 108N) + *dhps *437G ] was found in 82.3% of isolates. None of the samples contained quintuple mutations. There was no significant association between any particular genotype prevalence and patient age or sex.

Potential associations between alleles at different loci were evaluated, except for those whose prevalence was found to be fixed, or near fixation. Consequently, a non-random association was observed between the *pfdhfr *and *pfdhps *genes in the group of isolates collected during the first survey (2003/2004). Here, concomitant significant occurrence of wild-type/wild-type or mutant/mutant *pfdhfr*51/*pfdhps *437 alleles were observed among 29 out of 31 samples, whilst *pfdhfr *59/*pfdhps *437 occurred in 25 of the 33 cases successfully genotyped (Table 2).

**Table 2:** Significant associations between different alleles of the *Pfdhfr *e *Pfdhps *genes.

**Codons**	**Genotype**	**Number observed**	**Fisher's Exact Test (2-sided)** **(P- value)**
51 × 437 (N = 31)	N-A (wild-type)	8	P = 0.001 < 0.05
	I-G (mutant)	21	
59 × 437 (N = 33)	C-A (wild-type)	7	P = 0.001 < 0.05
	R-G (mutant)	18	

## Discussion

The present study evaluated mutation prevalence in the *Pfcrt*, *Pfmdr*1, *Pfdhfr *and *Pfdhps *genes among natural parasite populations of East Timor. Overall, the frequency of mutant alleles progressively increased from the first to second period, including those suggested to be involved in chloroquine resistance. These data is in contrast with previous studies where the frequency of the *Pfcrt *76T mutation decreased progressively with abolition of chloroquine for treatment for *P. falciparum *malaria [20,21]. The explanation for the persistence of the K76T mutation in East Timor *P. falciparum *populations may entail several factors. First, despite the discontinuation of CQ, the drug was still recommended for vivax malaria infections. Vivax malaria accounts for 20 – 40% of all malaria cases [7]. Furthermore, there is the problem of underestimation of mixed infections, as the diagnosis of malaria species in East Timor is made by conventional microscopy alone. Second, anti-malarial drugs are still widely available commercially in East Timor and most people use drugs acquired from parallel markets. The indiscriminate or inappropriate use and unsupervised drugs use for malaria infections is likely to be implicated in the maintenance of CQ-resistant parasites. Third, widespread use of quinine and amodiaquine in East Timor, which have been associated to certain extent to *pfcrt *76T and *pfmdr*1 86Y mutations [22,23], may impose positive selection, maintaining CQ resistance. Fourth, there has been extensive population movement between East Timor and West Timor-Indonesia, where malaria is highly endemic and CQ resistance levels are high. Thus, dissemination of resistant genotypes is likely to play an important role in maintaining CQ resistance in the region. Last, the reduced genetic diversity and lower recombination rates in south-east Asian parasites (when compared to Africa) may help maintain predominant genotypes even if mutations carry a fitness cost.

Point mutations in *pfmdr*1 N86Y and D1246Y, occasionally cited as potential contributors to chloroquine resistance [24] were also inspected. The *pfmdr*1 86Y allele was slightly predominant (56% prevalence) as previously observed both in Africa [25] and in some regions in Indonesia [26].

Since SP replaced CQ as recommended first line drug, the putative efficacy of SP was inferred by inspecting mutations in key codons of *P. falciparum dhfr *and *dhps *genes. Results indicated that mutation prevalence in those genes have increased steadily over a short period of time in contrast to an earlier study by [15]. Additionally, all isolates exhibited the *pfdhfr *108N mutation in combination with *dhfr *51I and/or *dhfr *59R mutations, indicative of increased levels of pyrimethamine resistance [24,27-29]. Also, triple mutants in the *Pfdhfr *gene (51I+59R+108N) accounted for 85.5% of all isolates further re-enforcing that high levels of pyrimethamine resistance are present [27,30].

The most common mutations polymorphism in the *pfdhps *gene was the 437G; only one case presented a 540E mutation. These observations are consistent with *pfdhps *437G being the first to emerge as result of pressure by sulpha drugs [11,31,32], which confers resistance to sulphadoxine in *P. falciparum *[17,33,34]. It was also verified that a high number isolates (82.3%) carried 4 mutations distributed among the *dhfr *and *dhps *genes {quadruple mutation: (51I + 59R + 108N + 437G)}, a pattern inferred in the presence of high levels of resistance to the pyrimethamine and in some cases, clinical resistance to SP [35].

*Pfdhfr *is present in chromosome 4 and *pfdhps *lies present in chromosome 8 of *P. falciparum*. Thus, both genes are physically distant and in conditions of normal transmission any particular haplotypes of each gene should be found randomly in human hosts. However, the present data highlights that resistance-associated alleles encoding both the *pfdhfr *51 and 59 mutants are non-randomly associated with *pfdhps *437 mutants, therefore indicating that both loci are under strong directional selection by sulphadoxine-pyrimethamine (SP), suggestive of selective advantage conferred by the presence of the two resistant alleles.

## Conclusion

This work suggests that SP resistance may already exist in the East Timor, and that the continuous use of the drug will contribute to higher patterns of inefficacy in the treatment of falciparum malaria. Nevertheless, these findings could be complemented with *in vivo *data as to reflect more closely the therapeutic efficacy of CQ and SP patterns in the epidemiological scenario. Persistence of CQ-resistant mutants even after supposed drug withdrawal re-enforces the need of its use against *P. vivax *exclusively. The association between *pfdhfr*51/*pfdhps*437 and *pfdhfr *59/*pfdhps *437 alleles indicates that these are undergoing concomitant positive selection.

## Competing interests

The authors declare that they have no competing interests.

## Authors' contributions

AA participated in the design of the study, conducted the field work, analysed and interpreted the data and contributed for the manuscript. APA, VR and PC, conceived and coordinated the study and drafted the manuscript.
